# Retraction Note: Aging exacerbates the brain inflammatory micro-environment contributing to α-synuclein pathology and functional deficits in a mouse model of DLB/PD

**DOI:** 10.1186/s13024-024-00762-4

**Published:** 2024-10-16

**Authors:** Michiyo Iba, Ross A. McDevitt, Changyoun Kim, Roshni Roy, Dimitra Sarantopoulou, Ella Tommer, Byron Siegars, Michelle Sallin, Somin Kwon, Jyoti Misra Sen, Ranjan Sen, Eliezer Masliah

**Affiliations:** 1 Laboratory of Neurogenetics, Molecular Neuropathology Section, National Institute On Aging, National Institutes of Health, Bethesda, MD 20892 USA; 2 Mouse Phenotyping Unit, Comparative Medicine Section, National Institute On Aging, National Institutes of Health, Baltimore, MD 21224 USA; 3 Laboratory of Molecular Biology and Immunology, National Institute On Aging, National Institutes of Health, Baltimore, MD 21224 USA; 4 Laboratory of Clinical Investigation, National Institute On Aging, National Institutes of Health, Baltimore, MD 21224 USA; 5 Immunology Program, Department of Medicine, Johns Hopkins School of Medicine, Baltimore, MD 21224 USA; 6 Division of Neuroscience, National Institute On Aging, National Institutes of Health, Bethesda, MD 20814 USA

**Retraction Note** **:** **Molecular Neurodegeneration 17, 60 (2022)**

**https://doi.org/10.1186/s13024-022-00564-6**

The Editors have retracted this article. After publication, concerns were raised regarding image overlap in the figures. Specifically:

• Fig. 3g ɑ-syn pff Striatum 1 and 2 appear to overlap;

• Fig. 3e Vehicle Neocortex 3, Amygdala 3 and Striatum 1 (aged mice) appear to overlap with Fig. 4a Vehicle Neocortex, Amygdala and Striatum CD3, respectively (young mice);

• Fig. 4a ɑ-syn monomer Neocortex and ɑ-syn pff Amygdala Iba-1 images appear to overlap;

• Fig. 4a Vehicle and ɑ-syn monomer Neocrtex GFAP images appear to overlap;

• Fig. 5c Vehicle Neocortex 1 and 3 appear to overlap;

• Fig. 5e and g all ɑ-syn pff Striatum images appear to be the same;

• Fig. 5e ɑ-syn pff Stiatum 1 and Amygdala 2 images appear to overlap;

• Fig. 5g Vehicle Striatum 1 and 3 appear to overlap;

• Similarly, there appear to be a number of cases of overlap between different samples or groups in the Supplementary figures.

The authors have stated that incorrect images were included in the article by mistake and provided the raw data for validation. However, further checks by the publisher found highly similar images between the raw data provided for different groups or experiments.

The Editors therefore no longer have confidence in the presented data.

The authors have agreed to this retraction, but not the wording of this retraction notice.

